# Supplementary material for: Neural Mechanisms of Acceptance and Commitment Therapy for Chronic Pain: A Network-Based fMRI Approach
Source: Front Hum Neurosci. 2021 Feb 5;15:587018. doi: 10.3389/fnhum.2021.587018 (PMC7892587; doi:10.3389/fnhum.2021.587018)
Supplement: Supplementary file 1 [file Table_1.pdf]

Supplementary Material

Supplementary Table 1. Power et al. 2011 nodes were used in graph analyses. Listed below are ROIs corresponding to the DMN, FPN, and SN.

Power ROI	X	Y	Z	Network	Region
74	-41	-75	26	Default mode	Occipital_Mid_L
75	6	67	-4	Default mode	Frontal_Mid_Orb_R
76	8	48	-15	Default mode	Frontal_Mid_Orb_R
77	-13	-40	1	Default mode	Lingual_L
78	-18	63	-9	Default mode	Frontal_Sup_Orb_L
79	-46	-61	21	Default mode	Temporal_Mid_L
80	43	-72	28	Default mode	Occipital_Mid_R
81	-44	12	-34	Default mode	Temporal_Pole_Mid_L
82	46	16	-30	Default mode	
83	-68	-23	-16	Default mode	
86	-44	-65	35	Default mode	Angular_L
87	-39	-75	44	Default mode	Angular_L
88	-7	-55	27	Default mode	Precuneus_L
89	6	-59	35	Default mode	Precuneus_R
90	-11	-56	16	Default mode	Precuneus_L
91	-3	-49	13	Default mode	Precuneus_L
92	8	-48	31	Default mode	Cingulum_Post_R
93	15	-63	26	Default mode	Precuneus_R
94	-2	-37	44	Default mode	Cingulum_Mid_L
95	11	-54	17	Default mode	Precuneus_R
96	52	-59	36	Default mode	Angular_R
97	23	33	48	Default mode	Frontal_Sup_R
98	-10	39	52	Default mode	Frontal_Sup_Medial_L
99	-16	29	53	Default mode	Frontal_Sup_L
100	-35	20	51	Default mode	Frontal_Mid_L
101	22	39	39	Default mode	Frontal_Sup_R
102	13	55	38	Default mode	Frontal_Sup_Medial_R
103	-10	55	39	Default mode	Frontal_Sup_L
104	-20	45	39	Default mode	Frontal_Sup_L
105	6	54	16	Default mode	Frontal_Sup_Medial_R
106	6	64	22	Default mode	Frontal_Sup_Medial_R
107	-7	51	-1	Default mode	Cingulum_Ant_L
108	9	54	3	Default mode	Frontal_Sup_Medial_R

109	-3	44	-9	Default mode	Frontal_Mid_Orb_L
110	8	42	-5	Default mode	Frontal_Mid_Orb_R
111	-11	45	8	Default mode	Cingulum_Ant_L
112	-2	38	36	Default mode	Frontal_Sup_Medial_L
113	-3	42	16	Default mode	Cingulum_Ant_L
114	-20	64	19	Default mode	Frontal_Sup_L
115	-8	48	23	Default mode	Frontal_Sup_Medial_L
116	65	-12	-19	Default mode	
117	-56	-13	-10	Default mode	Temporal_Mid_L
118	-58	-30	-4	Default mode	Temporal_Mid_L
119	65	-31	-9	Default mode	Temporal_Mid_R
120	-68	-41	-5	Default mode	
121	13	30	59	Default mode	
122	12	36	20	Default mode	Cingulum_Ant_R
123	52	-2	-16	Default mode	Temporal_Mid_R
124	-26	-40	-8	Default mode	ParaHippocampal_L
125	27	-37	-13	Default mode	ParaHippocampal_R
126	-34	-38	-16	Default mode	Fusiform_L
127	28	-77	-32	Default mode	Cerebelum_Crus1_R
128	52	7	-30	Default mode	Temporal_Mid_R
129	-53	3	-27	Default mode	Temporal_Mid_L
130	47	-50	29	Default mode	Angular_R
131	-49	-42	1	Default mode	Temporal_Mid_L
137	-46	31	-13	Default mode	Frontal_Inf_Orb_L
139	49	35	-12	Default mode	Frontal_Inf_Orb_R
**174**	-44	2	46	Fronto-parietal Task Control	Precentral_L
**175**	48	25	27	Fronto-parietal Task Control	Frontal_Inf_Tri_R
**176**	-47	11	23	Fronto-parietal Task Control	Frontal_Inf_Oper_L
**177**	-53	-49	43	Fronto-parietal Task Control	Parietal_Inf_L
**178**	-23	11	64	Fronto-parietal Task Control	Frontal_Sup_L
**179**	58	-53	-14	Fronto-parietal Task Control	Temporal_Inf_R
**180**	24	45	-15	Fronto-parietal Task Control	Frontal_Sup_Orb_R
**181**	34	54	-13	Fronto-parietal Task Control	Frontal_Mid_Orb_R
**186**	47	10	33	Fronto-parietal Task Control	Frontal_Inf_Oper_R
**187**	-41	6	33	Fronto-parietal Task Control	Precentral_L
**188**	-42	38	21	Fronto-parietal Task Control	Frontal_Mid_L
**189**	38	43	15	Fronto-parietal Task Control	Frontal_Mid_R
**190**	49	-42	45	Fronto-parietal Task Control	SupraMarginal_R
**191**	-28	-58	48	Fronto-parietal Task Control	Parietal_Sup_L

**192**	44	-53	47	Fronto-parietal Task Control	Parietal_Inf_R
**193**	32	14	56	Fronto-parietal Task Control	Frontal_Mid_R
**194**	37	-65	40	Fronto-parietal Task Control	Angular_R
**195**	-42	-55	45	Fronto-parietal Task Control	Parietal_Inf_L
**196**	40	18	40	Fronto-parietal Task Control	Frontal_Mid_R
**197**	-34	55	4	Fronto-parietal Task Control	Frontal_Mid_L
**198**	-42	45	-2	Fronto-parietal Task Control	Frontal_Mid_Orb_L
**199**	33	-53	44	Fronto-parietal Task Control	Parietal_Inf_R
**200**	43	49	-2	Fronto-parietal Task Control	Frontal_Mid_Orb_R
**201**	-42	25	30	Fronto-parietal Task Control	Frontal_Inf_Tri_L
**202**	-3	26	44	Fronto-parietal Task Control	Frontal_Sup_Medial_L
203	11	-39	50	Salience	Cingulum_Mid_R
**204**	55	-45	37	Salience	SupraMarginal_R
**205**	42	0	47	Salience	Precentral_R
**206**	31	33	26	Salience	Frontal_Mid_R
**207**	48	22	10	Salience	Frontal_Inf_Tri_R
**208**	-35	20	0	Salience	Insula_L
**209**	36	22	3	Salience	Insula_R
**210**	37	32	-2	Salience	Frontal_Inf_Orb_R
**211**	34	16	-8	Salience	Insula_R
**212**	-11	26	25	Salience	Cingulum_Ant_L
**213**	-1	15	44	Salience	Supp_Motor_Area_L
**214**	-28	52	21	Salience	Frontal_Mid_L
**215**	0	30	27	Salience	Cingulum_Ant_L
**216**	5	23	37	Salience	Cingulum_Mid_R
**217**	10	22	27	Salience	Cingulum_Ant_R
**218**	31	56	14	Salience	Frontal_Sup_R
**219**	26	50	27	Salience	Frontal_Mid_R
**220**	-39	51	17	Salience	Frontal_Mid_L

Supplementary Table 2. ACT Intervention, weekly ACT session topics and objectives.

	Session Topic	Session Objective	Homework Assignment
**Week 1**	Introductions & Foundations of Treatment	Examine pain duration and analyze past treatment	Creative Hopelessness & Mindfulness Practice: Monitor negative experiences/thoughts, and responses to those experiences throughout the week. Complete 3 five-minute mindfulness practice sessions
	Setting a Course for Treatment	Introduce behavioral model; in-vivo mindfulness practice	Check-in with yourself: Take 10-15 minutes each day to notice and observe.
**Week 2**	"Learning to Live" with Chronic Pain	Introduce/identify acceptance and values; in-vivo mindfulness practice	Values assessment: Identify personal values, rank importance and success
	Values and Action	Values clarification and goal setting; identify barriers; in-vivo mindfulness practice	Goals and action: Identify values and goals consistent with each value
**Week 3**	Urges, Thoughts, & Feelings	Defusion of threats/barriers; in-vivo mindfulness practice	Observing your actions: Track actions and observations throughout the week
	Action – Getting Your Feet Moving	Committed action; in-vivo mindfulness practice	Commit yourself to action: Track progress, difficulties, and experiences and review throughout the week
**Week 4**	Commitment	Acceptance, willingness, and barriers to willingness; in-vivo mindfulness practice	Mindfulness practice: Try new ways to practice mindfulness in daily life
	Lifelong Maintenance	Values clarification; dealing with relapses and setbacks; review treatment plan	Lifelong assignment: Continued values and goals assessment; mindfulness practice

Description of the ACT therapist training process can be accessed at:
Aytur, S., Campbell, C., Meier, S., et al. (2019). Broadening the scope of Acceptance and Commitment Therapy as a public health intervention for persons with chronic pain. Presented at the American Public Health Association annual conference, Philadelphia, PA, Nov. 5, 2019. https://apha.confex.com/apha/2019/meetingapp.cgi/Paper/453678

Supplementary Figure 1. ACT Intervention, structure of each ACT session.

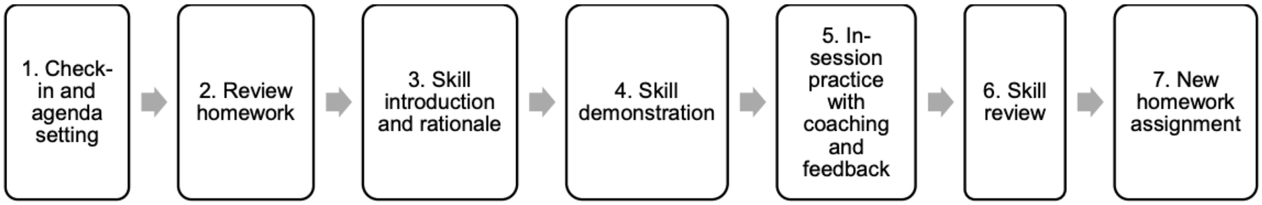

Supplementary Table 3. Behavioral assessments.

**Battery/Assessment**
Center for Epidemiologic Studies Depression Scale (CES-D; Radloff, 1977)
Brief Pain Inventory (BPI; Cleeland & Ryan, 1994)
Acceptance & Action Questionnaire (AAQ-II; Bond et al., 2011)
Chronic Pain Acceptance Questionnaire (CPAQ; Vowles et al., 2011)
Patient-Reported Outcomes Measurement Information System (PROMIS ®; Cella et al., 2010): Pain Interference, Pain Intensity, Pain Behavior
Quality of Life in Neurological Disorders (Neuro QoL; Cella et al., 2012) - Short Forms: Cognitive Function, Satisfaction with Social Roles and Activities, Stigma, Sleep, Positive Affect and Well-Being, Fatigue, Emotional and Behavioral Dyscontrol, Depression, Anxiety, Ability to Participate in Social Roles and Activities, Communication

Supplementary Table 4. Patient Characteristics.

**Patient ID**	**1**	**2**	**3**	**4**	**5**	**6**	**7**	**8**	**9**
**Age (years)**	66.33	38.33	61.08	62.08	20.49	53.54	45.22	60.13	21.1
**Chronic Pain Diagnosis/ Condition(s)**	fibromyalgia<br><br>trigeminal neuralgia; cervical spinal stenosis; lumbar spinal stenosis	fibromyalgia<br><br>diagnosed 2 yrs. after being in car-accident in 2007 (sustained injuries possibly resulting in condition)	chronic neck/shoulder/between shoulder pain; living at 5 (pain level); always has had issues throughout adulthood; injured at gym 1.5 yrs ago	lower back pain (injured 4 yrs ago); muscular, tight, spasms	Conradi Hunermann Syndrome	car accident (3 yrs ago), broken hip; has led to full body musculo-skeletal issues; spinal arthritis; nerve damage down left leg	fibromyalgia	fibromyalgia	Ehlers-Danlos syndrome; hyper-flexibility causes joints to be easily dislocated, muscle spasms
**Comorbid Conditions**	cataracts	food allergies	depression, anxiety	depression	none	anxiety, panic attacks, depression, PTSD	depression	depression, PTSD (military-related)	depression, ADD
**Occupation**	college professor; recently retired	former early educator (pre-school); now college student	college professor, part-time, semi-retired	college professor	college student	small business owner	college instructor	environment/ safety science company employee	recently graduated high school; planning to attend college
**Alternative treatments tried in the past**	Physical therapy (PT), pool exercises, mindfulness (many years ago; followed a book)	3 yrs ago - acupuncture; 2009 - chiropractic care	chiropractic care, acupuncture, therapeutic massage	therapeutic massage	dry needling	chiropractic care	none	PT, heated pool exercise	PT, heated pool exercise

**Surgeries**	right wrist - plates & screws; 2 rotator cuff repairs	none	none	none	multiple hip and spinal surgeries over past 15 years including: posterior spinal fusion- 2013; 2014 - translational calcaneal osteotomy; 2018 - translational calcaneal osteotomy & removal; 2018 - femoral head/neck osteo-chondroplasty, relative femoral neck lengthening, surgical hip dislocation	27 years ago - caesarian section; 20 years ago labrum tear - hip rebuild; 2 years ago - hip arthroscopy	caesarian-section	14 yrs ago - breast cancer	none
**Length of Pain (years)**	30	12	1.5	4	5	3.2	4.5	15	3
**Medications (opiates)** ***No new medications were started during the study period**	Tylenol #3 with Codeine	none	none	none	none	none	none	none	none
**BMI**	24.40	22.10	25.80	24.10	20.00	21.60	22.90	27.50	32.80
**Race/ ethnicity (1- white, 2- middle eastern)**	1	1	1	1	1	2	1	1	1
**Education (years)**	17.5	19	18	18	14	12	19	18	12
